# Isolated Lower Limb Weakness Following Hemorrhagic Stroke: A Case Report

**DOI:** 10.7759/cureus.38798

**Published:** 2023-05-09

**Authors:** Hannah L Price, Sam G Campbell

**Affiliations:** 1 Emergency Medicine, Dalhousie University, Halifax, CAN

**Keywords:** focal neurological deficit, ischemic and hemorrhagic stroke, adult emergency department, acute onset weakness, pure motor monoparesis, acute hemorrhagic stroke

## Abstract

Isolated limb weakness (monoparesis) has many possible etiologies. Although often assumed to be of a peripheral cause, it can be of central origin. This article describes a case from the Emergency Department of left lower limb weakness in a walk-in male patient on no medications, who had a 50-pack-year smoking history, type II diabetes, and asymptomatic atrial fibrillation. The patient had no history of previous episodes or trauma. His vitals were normal, and his speech and facial function were intact. The patient had full function of his upper limbs, no sensory deficits, and equal reflexes bilaterally. The singular clinical finding was decreased strength in the left leg compared to the right. Imaging revealed a right frontal intraparenchymal hemorrhage, which remained stable throughout his hospital admission. His muscle weakness was significantly improved upon discharge. In general, strokes can present with a variety of symptoms, which increase the risk of misdiagnosis. Monoparesis can be the singular sign of a stroke, and it is more common in the upper than the lower limbs.

## Introduction

Isolated limb weakness (monoparesis) is defined as decreased muscle strength in one limb. The differential diagnosis for monoparesis can include lesions to the brain, spinal cord, and affected limb. The first step in localizing a neurological lesion is to determine the pattern of deficits in order to refine the differential diagnosis. A non-exhaustive list of causes of monoparesis includes radiculopathy, myelopathy, myopathy, neuropathy, cerebral damage, ischemic lesion, and distal embolism [1]. Radiculopathy is the interference of nerve root function, which can cause pain, weakness, numbness, or tingling in the area innervated by the affected nerve root. Myelopathy is compression or injury to the spinal cord, which can cause loss of function or sensation below the level of the lesion, usually bilaterally. Myopathy is the disease of the muscle tissue affecting voluntary muscle movement, and it often presents as bilateral muscle weakness and cramping. Neuropathy is damage to one or more nerves caused by an injury or diabetes, typically resulting in numbness, tingling, muscle weakness, and pain. Brain abscess, hemorrhage, ischemic stroke, or tumor in the frontal lobe can cause muscle weakness or paralysis of the area controlled by the affected part of the cerebrum, which can be described by the homuncular organization of the motor cortex. Peripheral artery disease leading to acute thrombosis or distal embolism can result in paralysis, paresis, numbness, or pain.

It is important to be able to differentiate between central and peripheral lesions in order to provide the appropriate treatment [2]. On physical exam, some key factors to consider are the pattern of muscle groups affected; the presence or absence of atrophy, fasciculations, or spasticity; hyperactivity of deep tendon reflexes; and the presence of extensor plantar reflexes [2]. After the initial assessment, further testing may help refine the differential diagnosis, including a complete blood count (CBC), electrolytes, glucose, coagulation status (international normalized ratio (INR) and partial thromboplastin time (PTT)), and creatinine. These tests assess for disease states such as diabetes and hypercoagulability. An electrocardiogram (ECG) should be performed to look for cardiac arrhythmia, particularly atrial fibrillation, as it is associated with ischemic stroke. If a central cause is suspected, initial imaging should include magnetic resonance imaging (MRI) or unenhanced cranial computerized tomography (CT) to look for infarction or hemorrhage, with contrast added if there is suspicion of other space-occupying lesions. If there is a concern for metastatic brain lesions, a CT or chest x-ray may reveal a primary lung lesion.

## Case presentation

A 60-year-old male presented to the Emergency Department (ED) with an acute inability to bear weight on his left leg, which he had noticed standing up from the toilet nine hours prior to the ED presentation. He reported no numbness or other sensory changes. There was no history of trauma. He reported a 50-pack-year smoking history and denied any other recreational or prescribed substance use. He was diagnosed with type II diabetes mellitus 10 years previously. He also reported a history of asymptomatic atrial fibrillation. He denied a history of hypertension or dyslipidemia. He had previously been prescribed medications for diabetes management and aspirin for his atrial fibrillation, but he had stopped taking them five years previously. He was not taking any medications at the time of his ED visit. 

On examination, he appeared alert and oriented, with speech and facial function intact. He had a respiratory rate of 19/minute, SpO_2_ of 99% on room air, a pulse rate of 85/minute, a BP of 140/95 mmHg, and a temperature of 36.7°. His upper limbs had full strength (5/5) and sensation. The right leg had 5/5 strength. Left leg weakness of 3/5 was apparent throughout hip, knee, and ankle resisted motion tests. Bulk and tone were normal, with no fasciculation noted. Coordination was normal. Leg sensation was equal bilaterally. Tendon reflexes were brisk and symmetrical, apart from a Babinski equivocal on the left. 

His ECG showed atrial fibrillation. Blood work was normal and revealed a well-controlled Hb A1C level of 6.5%. There was no evidence of anemia. Platelet count, renal function, and electrolytes were all within normal range. His chest x-ray was clear.

**Figure 1:**
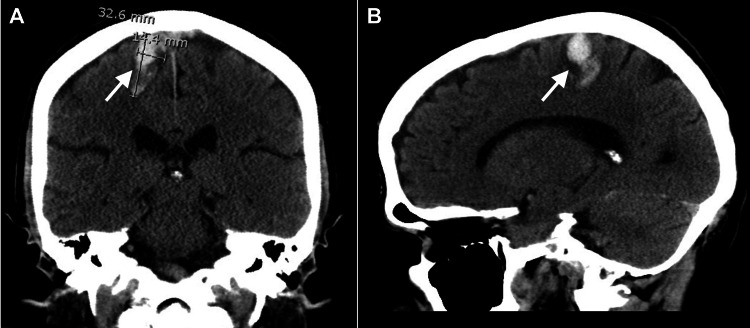
Initial unenhanced cranial computerized tomography scan in coronal (A) and sagittal (B) views

**Figure 2:**
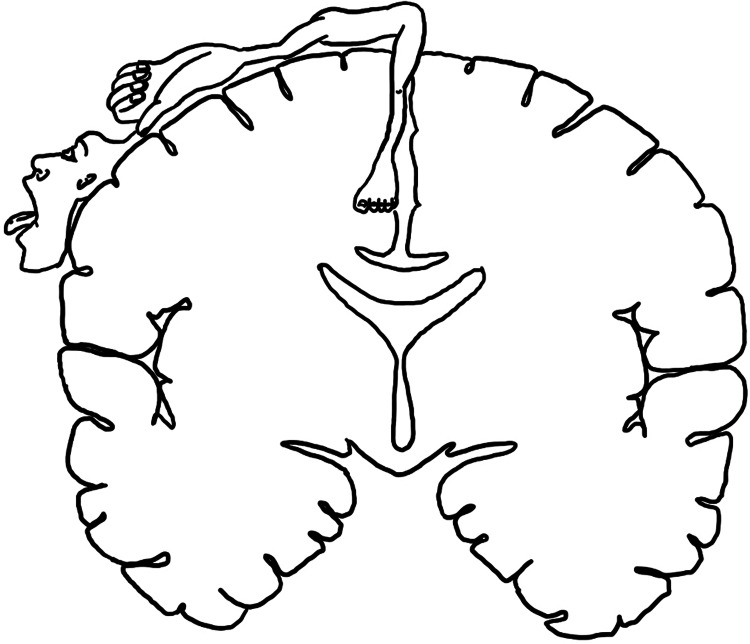
Proportional representation of the body in the primary motor cortex (homunculus) Image Credits: Hannah Price

The patient’s first unenhanced CT imaging showed an intraparenchymal hemorrhage in the right frontal lobe with vasogenic edema. The lesion measured 3.3 x 1.4 x 2.1cm with no significant mass effect or midline shift (Figures 1A, 1B). The position of the lesion corresponded to the lower limb territory in the primary motor cortex of the precentral gyrus, as described by the homunculus (Figure 2).

**Figure 3:**
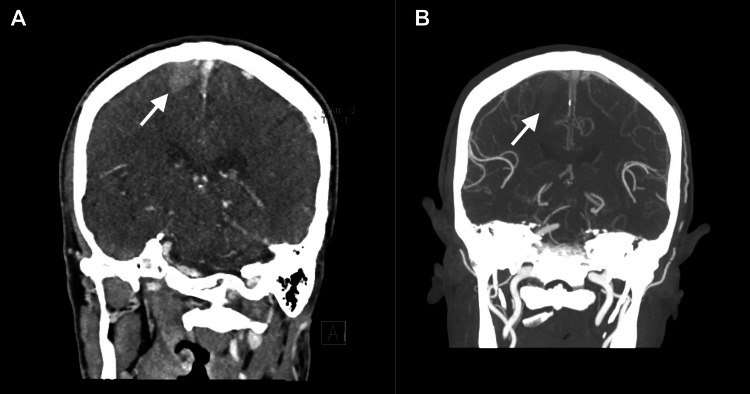
Cranial computerized tomography angiogram taken four hours after initial imaging in the coronal view (A) and with reconstruction (B)

A follow-up CT angiogram (Figures 3A, 3B), performed four hours after initial imaging, showed the intraparenchymal hemorrhage to be stable, as no spot sign was noted on the CT angiogram to suggest active arterial hemorrhage. There was no evidence of venous sinus thrombosis, vascular malformation, hydrocephalus, or underlying mass lesions. The intracranial arteries were widely patent, with no filling defects or aneurysms noted. The patient was diagnosed with a stable hemorrhagic stroke and admitted to the stroke unit for further observation.

**Figure 4:**
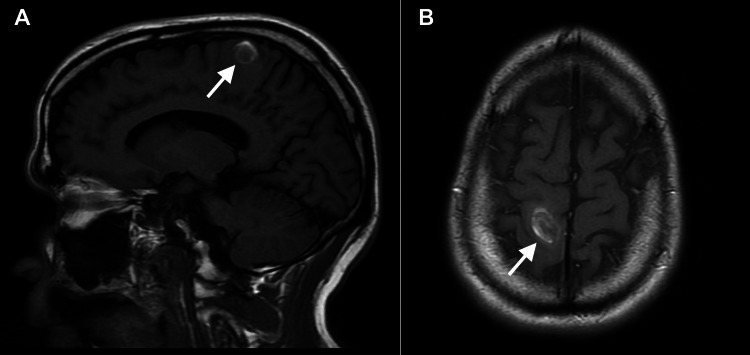
Cranial magnetic resonance imaging taken one week following presentation to the emergency department in sagittal (A) and axial (B) views

On the MRI performed a week later (Figures 4A, 4B), no other intracranial lesions or pathologic contrast enhancements were observed. Findings suggested that the intraparenchymal hemorrhage in the high right frontal lobe was the result of amyloid angiopathy. The lesion was stable at 2.2 x 1.5cm.

The patient’s weakness improved significantly over the next two weeks. He was discharged on anticoagulation therapy for his atrial fibrillation with aggressive risk factor control, including smoking cessation support and diabetes management with metformin and gliclazide. A three-month follow-up MRI was arranged.

## Discussion

Strokes can present with an array of symptoms, including paresis, dysphagia, dysarthria, visual field loss, and disturbances of hemisensory and visuospatial perception [3]. A purely motor deficit isolated to one limb can occur during a stroke, but it is uncommon [4]. Specifically, monoparesis occurs in approximately 4% of patients admitted with their first stroke [4]. The majority of strokes are ischemic (80%), and the remainder are hemorrhagic [3]. This stays true for strokes presenting with monoparesis, with a range of 3%-24% caused by hemorrhagic compared to ischemic strokes [1]. It has been stated that any lesion placed along the course of the corticospinal tract is capable of provoking monoparesis [1,5]. Furthermore, previous studies have stated that pure motor monoparesis is associated with cortical and subcortical infarcts [4]. The vascular territory damaged by a stroke in patients with pure monoparesis has been identified as superficial middle cerebral artery (MCA) in 48%, subcortical (anterior lenticulostriate) in 31%, brainstem in 8%, and anterior cerebral artery (ACA) in 8% [4].

When monoparesis does occur as the result of a stroke, upper limb paresis is the most common presentation, occurring in 63% of monoparesis stroke patients [4]. As isolated upper limb weakness is predominately caused by traumatic injury or compression to the radial, ulnar, or median nerves, these presentations are usually initially referred to orthopedics for consultation [6,7]. When brain imaging is conducted, upper limb monoparesis has been found to be caused by infarctions in the “hand knob” area on the precentral gyrus of the posterior frontal lobe [2,6]. These deficits predominantly occur from damage in the superficial MCA distribution [4] and represent less than 1% of all ischemic stroke patients [6]. Isolated facial weakness is the second most common presentation of monoparesis, occurring in 22% of monoparesis stroke patients [4]. Facial weakness most commonly occurs from a subcortical stroke [4]. Isolated lower limb weakness, which primarily occurs due to damage in the ACA territory, is the least common presentation of monoparesis at only 15% [4]. Although isolated lower limb weakness caused by a cortical infarction is rare and limited literature is available on the subject [5,8], it has been found to be caused by lesions in the primary motor cortex, medial to the hand knob area of the precentral gyrus [8]. This location appears consistent with the lesion noted by imaging for this case.

Although the most common vascular risk factors for a purely motor stroke are hypertension and diabetes [9], the only risk factor that has been shown to be associated with stroke-causing monoparesis is hypertension [4]. This is most likely due to the rarity of this type of presentation. While monoparesis following stroke is typically acute, it can be slow in onset, leading to misdiagnosis. The lack of additional clinical symptoms in monoparesis presentations provides challenges for determining the origin, so it is essential to include stroke on the differential when investigating a patient who presents with monoparesis. In addition, it is important to conduct an MRI after a negative CT scan, as it has been found that a cranial CT may miss the lesion in 40% of patients with monoparesis following a stroke since the lesion can be quite small [5].

Fortunately, patients with purely motor strokes have been shown to have fewer medical complications, lower in-hospital mortality rates, shorter duration of hospital stay, and higher frequency of symptom resolution at hospital discharge [9]. In particular, a stroke presenting as monoparesis has a more favorable outcome compared to strokes with more extensive motor deficits [4]. Some studies have found the recovery of almost normal function [2,7]. The research is scarce to understand why, but it is hypothesized that adjacent areas may support function for the damaged cortical area, thus resulting in less permanent dysfunction [10].

## Conclusions

Although strokes presenting with monoparesis are uncommon, it is crucial to consider the possibility of stroke when presented with a patient with an acute onset of purely motor monoparesis. This highlights the importance of in-depth patient history and physical examination to aid in the appropriate diagnosis. Despite limited literature available on the subject, monoparesis appears to more commonly be the result of an ischemic rather than hemorrhagic stroke and affects the upper more than the lower limbs. Therefore, this case presents an unusual situation with lower limb monoparesis caused by hemorrhagic stroke. Fortunately, the outcome for monoparesis strokes is favorable compared to those with more extensive motor or sensory deficits.
